# Crazy paving pattern

**DOI:** 10.1002/ccr3.860

**Published:** 2017-02-21

**Authors:** Rajanshu Verma, Mamoun Abdoh

**Affiliations:** ^1^ Department of Hospital MedicineAugusta HealthFishersvilleVirginiaUSA; ^2^ Borgess Pulmonary & Sleep MedicineKalamazooMichiganUSA

**Keywords:** Crazy paving pattern, septal interlobular thickening, T‐cell lymphoma

## Abstract

Crazy paving pattern is the name given to characteristic polygonal pattern of interlobular thickening on computed tomography (CT scan) of the lung, which results from accumulation of collagen, interstitial fluid, cell, or other pathology and may be seen in multiple clinical conditions including metastatic cancer as described in this case.

A 63‐year‐old African American man presented with two‐week history of progressive dyspnea on exertion, dry hacking cough, Raynaud phenomenon, night sweats, and decreased appetite. He had 30 pack‐year history of smoking. Lactate dehydrogenase was elevated at 1563 U/L. CT scan of the chest showed “crazy paving pattern” with extensive ground‐glass opacities with superimposed interlobular septal thickening (see Fig. 1, Video S1). An open lung biopsy showed small irregular CD3+, CD5+, CD30+, CD20‐, and ALK‐1 lymphoid cells consistent with unspecified peripheral T‐cell lymphoma.

**Figure 1:**
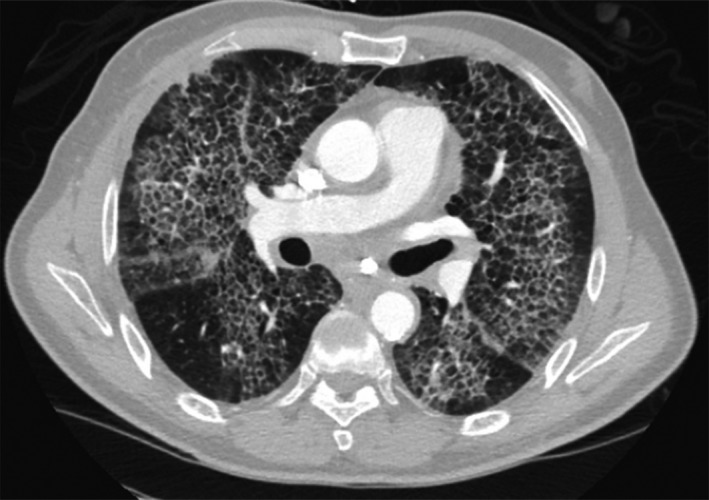
CT scan showing characteristic “crazy paving pattern” with polygonal honeycomb‐like interlobular septal thickening.

Question: Which other conditions can have similar presentation on CT chest?

Crazy paving pattern may be seen in pulmonary alveolar proteinosis, lipoid pneumonia, pneumocystis jiroveci pneumonia, sarcoidosis, nonspecific interstitial pneumonia, silicosis, cryptogenic organizing pneumonia, adult respiratory distress syndrome, and others 1, 2.

## Conflict of Interest

No source of funding or conflict of interest.

## Authorship

MA and RV: involved in taking care of the patient, collected clinical data, extracted radiology images, and prepared, edited, and approved the final manuscript.

## Supporting information


**Video S1.** Multi‐level axial chest CT scan cine sequence showing extent of crazy paving pattern.
